# Optimizing Fertility in Primary Ovarian Insufficiency: Case Report and Literature Review

**DOI:** 10.3389/fgene.2021.676262

**Published:** 2021-06-23

**Authors:** Kensuly C. Piedade, Hillary Spencer, Luca Persani, Lawrence M. Nelson

**Affiliations:** ^1^Mary Elizabeth Conover Foundation, Inc, McLean, VA, United States; ^2^Vanderbilt University Medical Center, Nashville, TN, United States; ^3^Department of Medical Biotechnology and Translational Medicine, University of Milan, Milan, Italy; ^4^Department of Endocrine and Metabolic Diseases, Istituto Auxologico Italiano, Milan, Italy

**Keywords:** primary ovarian insufficiency, pregnancy, luteinized follicles, hormone replacement therapy, intrauterine insemination

## Abstract

Primary ovarian insufficiency (POI) is a clinical spectrum of ovarian dysfunction. Overt POI presents with oligo/amenorrhea and hypergonadotropic hypogonadism before age 40 years. Overt POI involves chronic health problems to include increased morbidity and mortality related to estradiol deficiency and the associated osteoporosis and cardiovascular disease as well as psychological and psychiatric disorders related to the loss of reproductive hormones and infertility. Presently, with standard clinical testing, a mechanism for Overt POI can only be identified in about 10% of cases. Now discovery of new mechanisms permits an etiology to be identified in a research setting in 25–30% of overt cases. The most common genetic cause of Overt POI is premutation in *FMR1*. The associated infertility is life altering. Oocyte donation is effective, although many women prefer to conceive with their own ova. Surprisingly, the majority who have Overt POI still have detectable ovarian follicles (70%). The major mechanism of follicle dysfunction in Overt POI has been histologically defined by a prospective NIH study: inappropriate follicle luteinization due to the tonically elevated serum LH levels. A trial of physiologic hormone replacement therapy, clinically proven to suppress the elevated LH levels in these women, may improve follicle function and increase the chance of ovulation. Here, we report the case of a woman with Overt POI diagnosed at age 35 years. To attempt pregnancy, she elected a trial of intrauterine insemination (IUI) in conjunction with follicle monitoring and physiologic hormone replacement therapy. She conceived on the eighth cycle of treatment and delivered a healthy baby. Our report calls for a concerted effort to define the best methods by which to optimize fertility for women who have POI.

## Introduction

The diagnosis of primary ovarian insufficiency (POI) needs to be considered in the differential diagnosis for any woman of reproductive age who presents with infertility developing at <40 years of age. The disorder is a clinical spectrum of impaired ovarian function. Overt POI presents clinically as oligo/amenorrhea in association with menopausal level serum FSH values. Biochemical POI presents with infertility, regular menstrual cycles, and elevated cycle day 3 serum FSH levels. Occult POI presents with infertility, regular menstrual cycles, normal cycle day 3 serum FSH values, and a poor response to exogenous gonadotropin stimulation (Nelson, 2009).

In this case report, we describe a woman who presented with oligo/amenorrhea at age 29 years. At age 35, she was given a diagnosis of Overt POI. She conceived and delivered a healthy child by employing follicle monitoring in conjunction with physiologic hormone replacement therapy (P-HRT) and intrauterine insemination. The P-HRT was employed to suppress elevated serum LH levels in order to avoid premature and inappropriate follicle luteinization (Nelson et al., 1994). As a rare disorder, POI puts many clinicians at a loss on how to manage Overt POI and a lack of appreciation for: (1) the pathophysiology of follicle dysfunction in Overt POI (Nelson et al., 1994), (2) the many benefits bestowed by providing P-HRT (Popat et al., 2014), and (3) the possibility of pregnancy in this condition (Fraison et al., 2019).

## Case Report

In January 2012, a previously healthy 29-year-old woman physician presented with a 7-month history of oligo-amenorrhea and a noticeable increase in fatigue and sleepiness despite adequate sleep hygiene. She had no vasomotor symptoms or night sweats. Laboratory evaluation revealed normal thyroid function and a markedly elevated serum FSH level of 37.6 mU/ml (normal follicular phase range 2.5–10.2). In February 2012, repeat laboratory examinations revealed serum FSH at 8.7 mU/ml, LH at 4.8 mU/ml (normal 2–15), estradiol at 132 pg/ml (normal 30–190), and testosterone at 11 ng/dl (normal range: 25–125). At age 31, she began taking the oral contraceptive. In August 2017, at age 35, she consulted a reproductive endocrinologist to discuss her desire to have a child. At that time, 2 weeks after stopping the oral contraceptive, her FSH was 19.4 mu/ml, AMH 0.2 ng/ml, and her estradiol was 52 pg/ml. She was given a diagnosis of Overt POI. Her POI was idiopathic. She had a normal 46,XX karyotype, negative genetic testing for the fragile X mental retardation 1 (*FMR1*) premutation (29/32 CCG repeats), negative 21 hydroxylase antibody testing (<1 U/ml), and small ovaries on ultrasound examination. A bone density measurement was normal. A saline sonogram revealed a small endometrial polyp, which was removed by hysteroscopy. Consultants suggested egg donation as her best option to have a child and advised IVF and oocyte preservation were not options for her.

Here is how the patient describes her care experience after seeing several consultants:

“After my misdiagnosis and then difficulty accessing complete care for POI, I didn't know how to balance being a “good patient” and advocating for myself. I felt utterly dependent and distrusting at the same time. It made me sad that I couldn't find a physician to care for me the way I do for my own patients. I've been told that this should be humbling and help give me more empathy for my patients. I'm tired of being humbled. We – women with POI – deserve better. We deserve doctors who are informed about our condition, who know the limits of their knowledge, and who are not too proud to seek expertise.”

The patient thus sought out the assistance of a health coach board certified by the National Board for Health and Wellness Coaching (National Board for Health and Wellness Coaching, n.d.). She wanted to learn how to better advocate with her clinician regarding her specific needs as a woman living with Overt POI. The health coach also helped her to “more confidently make decisions regarding her treatment plan” and “to better process and deal with the inherent stresses of fertility treatments.” She elected to attempt conception with her own ova rather than using donor ova. She found a clinician willing to work with her special care needs.

In October 2018, while continuing the NIH P-HRT regimen to suppress LH levels, she conceived on the eighth cycle of intrauterine insemination (IUI) using donor sperm. On the NIH P-HRT regimen, her menstrual cycle day 3 serum LH level was in the normal range at LH 6.93 mIU/L. Her clinician monitored follicle development by serial transvaginal ultrasound, and the woman monitored urinary LH measurements. After the eighth cycle on this regimen, her home pregnancy test showed positive on day 13 post-IUI. This was confirmed by serial serum beta HCG measurements. The pregnancy was uncomplicated until at 35 weeks gestation when she developed gestational hypertension. There was concern also for intrauterine growth restriction. Due to ongoing concerns, her care team induced labor. The baby was born at 37 weeks and 4 days gestation. The neonatal course was complicated by being small for gestational age, with associated hypothermia, hypoglycemia, and hyperbilirubinemia. The baby was discharged home in good condition on the third day of life.

## Life Expectancy and Quality of Life

Importantly, convincing evidence has demonstrated women who have Overt POI in fact have a shorter life span. This is likely related to the long-term morbidity of chronic estradiol deficiency. A 2016 systematic review and meta-analysis addressed this issue. The report included an analysis of seven prospective studies producing nine articles. (Tao et al., 2016) the report showed women who have Overt POI as compared with controls have: (1) a higher risk of death from all causes and (2) a higher risk of death from ischemic heart disease. The study found no significant association with all-cancer mortality (Tao et al., 2016). If indeed the increased mortality is related to estradiol deficiency, one would expect a well-controlled prospective natural history study which provides physiologic HRT to improve their life expectancy. Importantly, an NIH prospective controlled study showed P-HRT restored the POI-related reduced bone density to normal by the end of 3 years (Popat et al., 2014). The NIH P-HRT regimen restored regular menses, relieved symptoms of estradiol deficiency, and was well-tolerated.

## Quality of Care

Most women who have Overt POI are not satisfied with the quality of care provided by their clinician (Groff et al., 2005). Overall, 71% were displeased with how their clinician informed them of the diagnosis. Most (89%) experienced moderate to severe emotional distress at the time. The degree of emotional distress was more severe in those women who were most dissatisfied with how they were told the diagnosis. The women reported several factors which helped then to cope with the diagnosis. These included getting thorough and accurate information about the disorder from the clinician, support of others, and spirituality. Most women find the diagnosis of Overt POI to be difficult to understand and emotionally traumatic. The findings suggest that clinicians can reduce the level of distress experienced by these women by better informing them about the diagnosis. Patients perceive a need to spend more time with clinicians and a need for more complete information about Overt POI (Groff et al., 2005).

## Emotional Health

Prospective controlled study has demonstrated women who have Overt POI as compared with controls have more symptoms of depression and anxiety, lower positive affect, and higher negative affect (Davis et al., 2010). In a controlled study, illness uncertainty was a significant independent risk factor associated with anxiety. Stigma was also a significant independent risk factor associated with depression. Goal reengagement was a significant and independent factor associated with positive affect. The findings suggest clinicians could improve the emotional well-being of their patients with Overt POI by: (a) informing them better about their condition, (b) educating to help them to feel less stigmatized by the disorder, and (c) assisting them in developing realistic goals with regard to family planning as well as other goals.

Social support is an important factor in quality of life. A prospective and controlled study demonstrated women who have Overt POI perceive less social support and greater stigma than control women (Orshan et al., 2009). Patients who perceived more social support in their life also had greater self-esteem. Marital status, whether or not the woman had children, or how long ago the diagnosis was established did not affect these findings. Overt POI is a life-altering diagnosis and strategies to improve social support and self-esteem are important considerations in reducing the emotional suffering that accompanies Overt POI.

Another important issue involves coping with the diagnosis of Overt POI. What is the evidence about the coping strategies women who have Overt POI? How do they address the associated emotional sequelae and behavioral health effects of the disorder? The NIH conducted a longitudinal study to address these questions (Driscoll et al., 2016). Importantly, analysis of the data identified a single stand-alone coping strategy which explained the outcome in affect at the end of 1 year. Avoidance is a coping strategy in which the patient refuses to acknowledge stress. Avoidance explained the association between the baseline vulnerability and the degree of distress measured at 12 months. Coping by avoidance has been linked to poorer psychosocial adaptation according to a systematic review published in 2019 (Livneh, 2019). The use of avoidance coping is largely ineffective when confronting stressful life events and health-related conditions. Furthermore, the reliance on avoidance strategies have been linked to poorer adaptation in a wide range of disorders (Livneh, 2019). Interventional studies targeted to reduce avoidance in women who have Overt POI are indicated.

One specialized area of particular concern is the emotional and behavioral health of teens who get a diagnosis of Overt POI. This diagnosis is both emotionally difficult and confusing not only for young girls but also their families. Parents require assistance to help their daughters understand the diagnosis. They need guidance to help the child maintain healthy growth and development. One published report provides a starting point on this path for parents and clinicians. A handout has been published entitled “Tips and Tools for Talking: Helping Your Daughter Understand Primary Ovarian Insufficiency” (Covington et al., 2011). Importantly, commonly the diagnosis of POI is delayed in teens. There is an assumption in teens that irregular menses are common, and therefore not in need of investigation. A family systems approach is an important component to the management plan for teens and their parents. Such an approach must incorporate mental health support. Ideally, a teen with Overt POI should have patient-centered and integrated comprehensive approach provided by a primary care clinician. The primary care clinician needs support from a multidisciplinary team (Gordon et al., 2015).

## Ovarian Function

Despite having oligo/amenorrhea and menopausal level serum FSH levels, 73% of women with Overt POI, nevertheless, have ovarian follicles remaining in the ovary (Nelson et al., 1994; Hubayter et al., 2010). Overt POI is ovarian function which is intermittent and unpredictable and may persist for many years. Graafian follicles grow in response to the high FSH levels, yet their function is impaired by the high serum LH levels. The high LH levels induce inappropriate luteinization and thus prevent normal follicle function and ovulation. Weekly blood sampling and sonography in 65 women with confirmed Overt POI revealed 50% demonstrated ovarian follicle function as defined by a serum estradiol >183 pmol/L (50 pg/ml) during 4 months of observation (Nelson et al., 1994). Importantly, 16% of these women achieved an ovulatory serum progesterone level as defined by >9.5 nmol/L (3.0 ng/ml).

**Figure 1:**
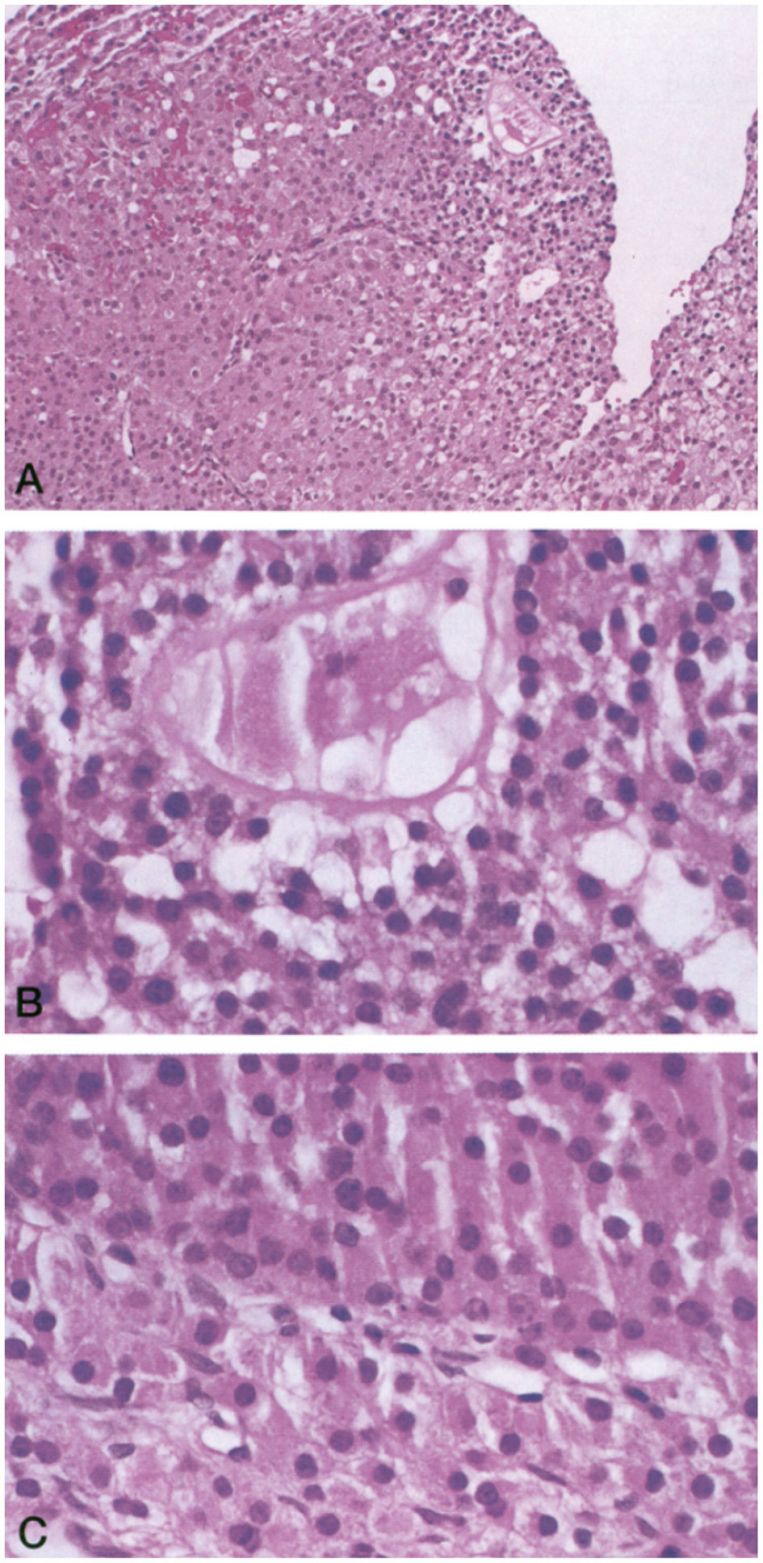
**(A)** Luteinized follicle containing a structure suggestive of an ovum undergoing degeneration (hematoxylin and eosin stain; magnification, ×150). **(B)** Higher magnification of the putative ovum (hematoxylin and eosin stain; magnification, ×250). **(C)** Higher magnification of the same follicle showing luteinized cells characterized by their larger size, abundant eosinophilic cytoplasm, and prominent nucleus (hematoxylin and eosin stain; magnification, ×250).

**Figure 2:**
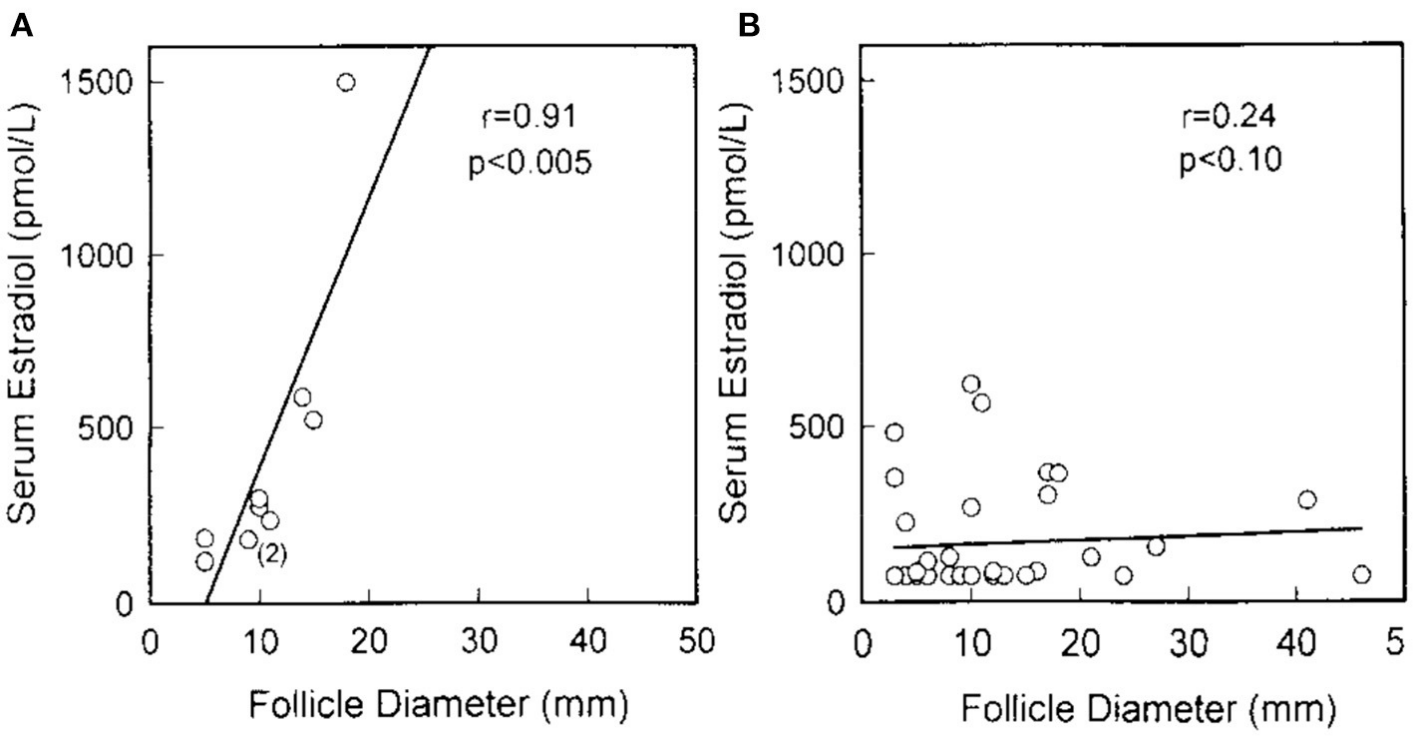
Serum estradiol correlated with maximum follicle diameter in normal women but not in women with Overt POI. **(A)** Each point represents the findings in 1 of 10 normal women with regular menses examined during the follicular phase. Two congruent points are noted by (2). **(B)** Each point represents the findings in a patient with overt POI who had an ovarian follicle detected by sonogram (37 sonograms in 27 patients). There are 8 congruent points (Nelson et al., 1994).

In a 1994 histologic study of ovarian follicle function, the NIH POI research team biopsied antral follicles in six women who had Overt POI. They demonstrated luteinized Graafian follicles in every case (six of six, 95% confidence limit 60%). Inappropriate luteinization of Graafian follicles is thus the major pathophysiological mechanism of follicle dysfunction in patients who have Overt POI (Figure 1) (Nelson et al., 1994). By sonography, the NIH team found antral follicles in over 40% of women (27 of 65). When an antral follicle was present serum estradiol was significantly greater (Nelson et al., 1994). The follicles were dysfunctional in these women, however. Women with Overt POI had poor correlation between follicle diameter and serum estradiol as compared to controls (Figure 2).

**Figure 3:**
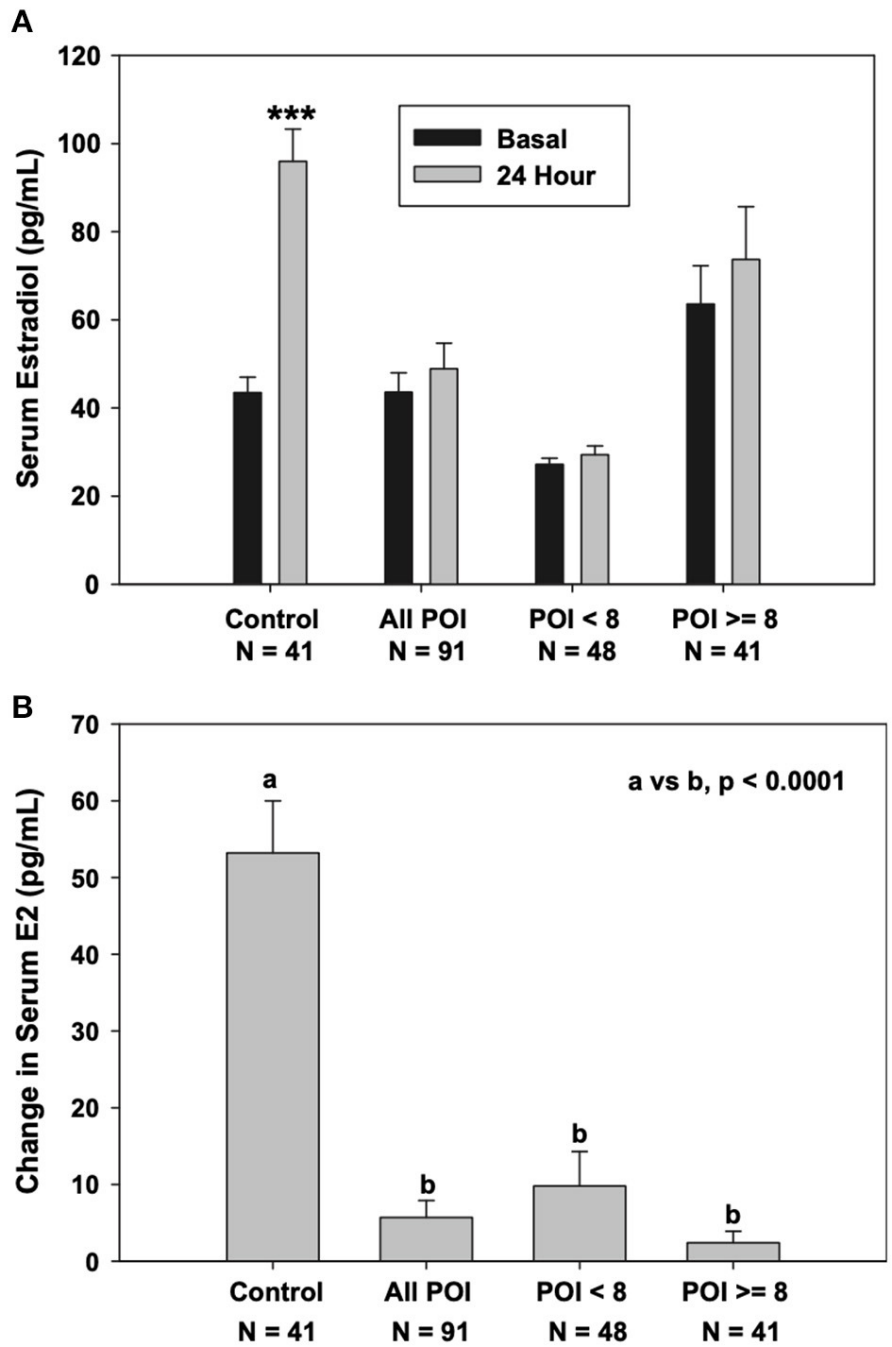
Serum estradiol response to stimulation with 300 IU FSH. **(A)** Control women, women with Overt POI, and women with overt POI segregated by the absence or presence of an antral follicle 8 mm in diameter or greater (^***^P < 0.0001 vs. baseline). **(B)** Change in serum estradiol levels at 24 h.

In 2010, the NIH POI research team also developed an ovarian stimulation test (OST). The aim was to assess ovarian follicle function in response to FSH stimulation in women confirmed to have spontaneous 46,XX Overt POI (Hubayter et al., 2010). The team employed a case-control study with a nested prospective cohort. They administered the OST to 97 women who had Overt POI and had taken no HRT for the prior 2 weeks. The control group was 42 regularly menstruating women who were in the mid-follicular phase of the menstrual cycle. The OST consisted of a single injection of 300 IU hrFSH and monitoring of the change in serum estradiol levels 24 h later. Antral follicles >3 mm was detected in 73% (69/95) of women with Overt POI. Maximum follicle diameter correlated significantly with both serum estradiol and progesterone levels. Patients who had a follicle with a maximum follicle diameter ≥8 mm had significantly lower FSH and LH levels. They also had significantly higher serum estradiol and progesterone levels. In women with Overt POI, the serum estradiol levels did not significantly increase after FSH administration despite the presence of an antral follicle ≥8 antral and mm. In contrast with control women, their serum estradiol levels increased significantly in response to FSH (Figure 3) (Hubayter et al., 2010).

The case reported herein is consistent with the following hypothesis: by employing the NIH P-HRT regimen to suppress LH levels in women who have Overt POI one could prevent follicle luteinization, restore follicle function, permit ovulation, and increase the chance of pregnancy. The evidence that 70% of women with Overt POI have antral follicles on ultrasound examination provides convincing evidence against the hypothesis that downregulation of FSH receptors is the mechanism of follicle dysfunction in these women. Interestingly, now two different lines of evidence come together to confirm the pathogenesis of follicle dysfunction in Overt POI. The two lines of evidence are (1) evidence of significant progesterone secretion by antral follicles ≥8 mm in women with Overt POI and (2) published evidence demonstrating histologically that follicle luteinization is the major mechanism of abnormal follicle function in this condition (Nelson et al., 1994). Prospective controlled investigation designed to prevent follicle luteinization and improve ovulatory function and fertility in these women who have Overt POI is clearly indicated.

## Physiologic Hormone Replacement Therapy

The estradiol deficiency of Overt POI is directly related to the associated health complications. Unfortunately, 52% of patients with Overt POI either never begin P-HRT at diagnosis, begin P-HRT many years later, and/or stop using P-HRT use before age 45 (Hipp et al., 2016). Unfortunately, findings regarding detrimental effects reported in the Women's Health Initiative (WHI) trial inappropriately causes many clinicians to refrain from prescribing hormone therapy for young women with Overt POI (Sullivan et al., 2016). The WHI was a study of older postmenopausal women, not young women who have Overt POI. It showed multiple increased health risks with hormone therapy. These included increased risk breast cancer, stroke, and cardiovascular disease (Rossouw et al., 2002). This is unfortunate because the clinical situation markedly differs. The clinical situation in young women with Overt POI is indeed pathological (an abnormal state of estradiol deficiency compared with women of the same age). Menopause is a normal state for older women. In women with Overt POI, the term P-HRT accurately describes the clinical purpose. The prescribed hormones in this case replace the hormones that would normally be produced by young women (Sullivan et al., 2016). P-HRT is now considered standard of care for women with Overt POI (Committee Opinion No. 698: hormone therapy in primary ovarian insufficiency, 2017). It is generally recommended to continue P-HRT until age 50 (the average age when natural menopause occurs).

**Figure 4:**
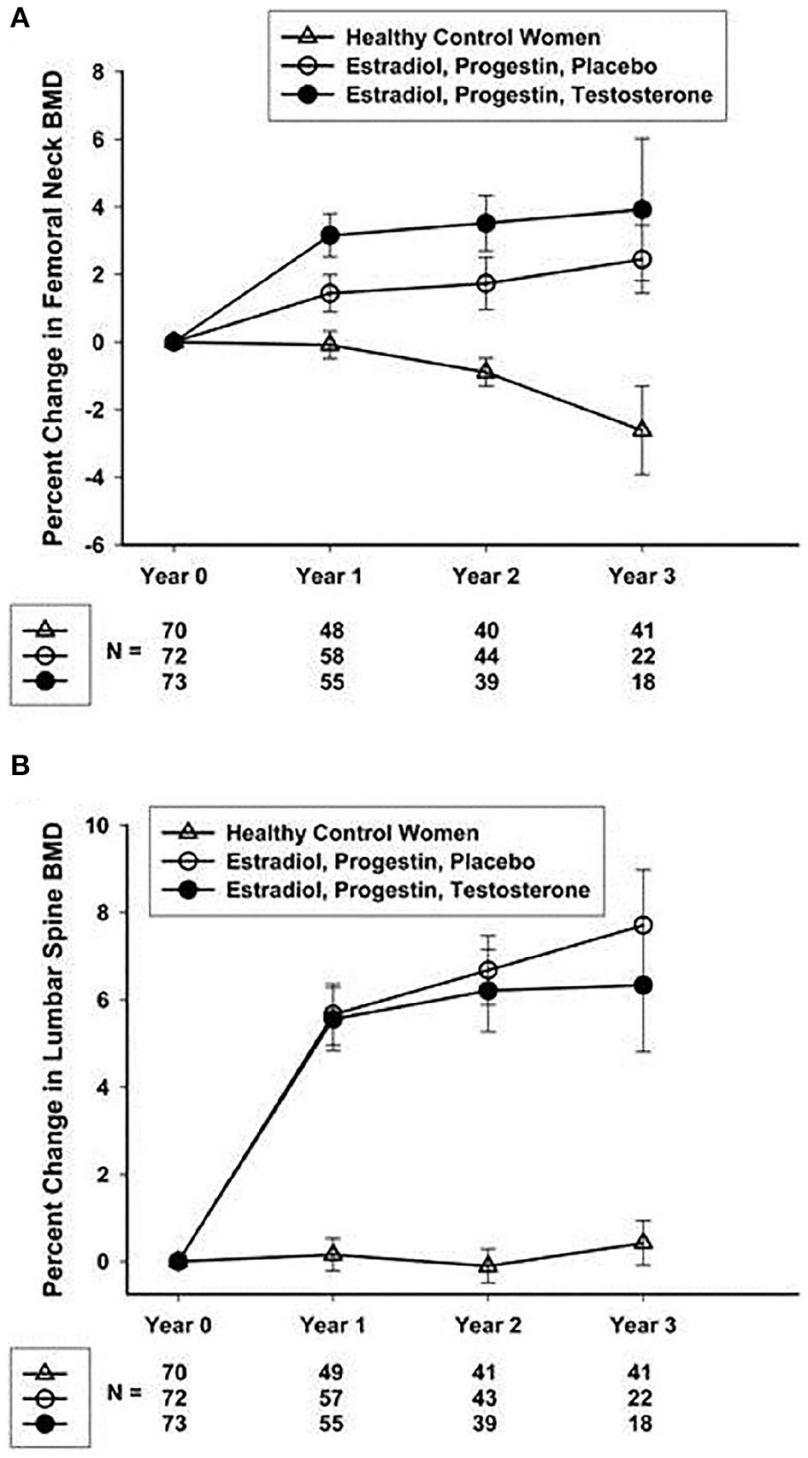
**(A)** Mean (SEM) percentage change from screening in the femoral neck BMD. **(B)** Mean (SEM) percentage change from screening in the lumbar spine BMD (Popat et al., 2014).

To test the hypothesis that P-HRT is an effective clinical approach, the NIH Intramural Research Program designed a prospective randomized controlled trial in young women who had Overt POI (Popat et al., 2014). The study lasted 3 years. The treatment employed physiologic estradiol replacement (transdermal estradiol 100 μg/day) and cyclic progestin (oral medroxyprogesterone 10 mg daily for 12 days/month). Importantly, this P-HRT resulted in lumbar spine and femoral neck BMD increasing significantly. In fact, by the end of the study, bone mineral density had increased to such a degree their BMD did not differ significantly from the control women who participated (Figure 4). Transdermal physiologic testosterone replacement provided no significant additional benefit to BMD (Popat et al., 2014). The NIH P-HRT regimen was well-tolerated by the study participants.

The ideal would be to mimic normal ovarian function as a hormone replacement strategy. An artificial ovary could theoretically be designed to employ a vaginal ring. This could deliver a steady parenteral release to mimic endogenous ovarian hormone production similar to a normal menstrual cycle. The transdermal patch and the vaginal ring that deliver 0.10 mg of estradiol per day are a fundamental and elegant step in this line of thinking. The design mimics the daily ovarian production rate of estradiol to put average serum estradiol levels of in the range of 100 pg/ml. This matches the average estradiol level across the menstrual cycle of control women who have normal ovarian function (Mishell et al., 1971). Transdermal and transvaginal routes of administration avoid the first pass effect of the liver (which occurs with orally delivered estrogen) by delivering hormones directly into the blood (Canonico et al., 2008). A systematic review concluded oral estrogen increases the risk of venous thromboembolism as compared with estradiol delivered transdermally (Mohammed et al., 2015). Importantly, in postmenopausal women, the relative risk of stroke is greater with oral estrogen compared with transdermal estradiol (Renoux et al., 2010).

The NIH study on P-HRT employed medroxyprogesterone acetate as the progestin. The NIH POI multidisciplinary team of clinicians and scientists had concerns regarding the use of oral micronized progesterone. They found inadequate evidence in the literature to demonstrate effectiveness in protecting the endometrium when used with full replacement doses of estradiol. Also of significant consideration, medroxyprogesterone acetate given orally is protected from metabolism by the liver by the addition of a simple medroxy group to the pure progesterone molecule. This agent has been in use for decades and has a wonderful safety record. In considering the possible use of oral micronized progesterone as part of the regimen, the NIH multidisciplinary team had significant concerns regarding the large number of metabolites resulting from the first pass effect on the liver (Sullivan et al., 2016). Some women who develop Overt POI in their younger years will be taking P-HRT for decades. The potential longer-term adverse health effects of these progesterone metabolites after exposure for decades are completely unknown. Another concern is the potential for adverse effects of these multiple progesterone metabolites on fertility and the developing embryo.

## Fertility

Pregnancy may occur in women who have POI in the absence of any medical intervention (Soave et al., 2013). The authors of a 2019 systematic review emphasized this important point, “To date no clinical test can determine the potential for conception in patients with POI.” (Fraison et al., 2019). Also, the authors warn clinicians to be prudent and not to be hasty in claiming absolute sterility when counseling women who have the diagnosis of Overt POI. The authors suggest an approach which encourages patients to take their time deciding about ova donation rather than rushing the decision. Some women are uncomfortable with ova donation and desire a chance to have a pregnancy with their endogenous ova. As in the case reported herein, many women who live with Overt POI do indeed wish to have this chance to conceive with their own ova before moving forward to egg donation (Fraison et al., 2019).

This 2019 systematic review assessed fertility in women who have Overt POI. The authors searched electronic databases for the years 2000 to 2018 (Fraison et al., 2019). They included 15 studies and found pregnancy rates from 2.2 to 14.2%. The average age of patients who got pregnant was 30 years. The authors report this evidence supports a conclusion that oocyte quality in women who have Overt POI is likely normal.

As noted above, many young women find the diagnosis of Overt POI emotionally devastating. Many find it difficult to grieve their infertility and employ the regrettable course of avoidance coping (Driscoll et al., 2016). This makes these women extremely vulnerable to misguided approaches to restore fertility, some quite expensive, emotionally draining, and not based on credible evidence. Some women will request treatment with agents to induce super ovulation despite having been informed of the minimal chance of success (Fraison et al., 2019). It is difficult to justify using ovarian stimulation protocols to improve fertility in this population. As noted above, results of two NIH prospective studies to assess ovarian follicle function in these women have demonstrated conclusively: follicles in women who have Overt POI are not capable of responding to FSH stimulation due to their luteinized state (Nelson et al., 1994; Hubayter et al., 2010).

The case presented herein raises an important question. How to proceed to optimize fertility in the face of Overt POI for those women and partners who do not pursue oocyte donation as a solution to their infertility? Many women and their partners find it very difficult to accept the idea of not passing their DNA on to their child. This is a bridge too far for some women and partners. Most donor oocyte programs offer pretreatment counseling to optimize the mental health of the child and family. Some will decide against using donor oocytes. In addition, ova donation is not always readily available. Many countries do not permit ova donation. Other countries do not permit payment to prospective donors, which severely limits availability of this approach. Importantly, questions still remain about the mental health of donor-conceived offspring. It is likely that many parents do no inform their donor conceived children about their origin (Bracewell-Milnes et al., 2018; Fraison et al., 2019).

As in any preconception situation, planning and preparing for pregnancy are important even if the chances of conceiving are limited. A visit to the care team is in order to: (1) assess any other medical conditions; (2) review medications; (3) review vaccinations; (4) review family history; (5) begin taking folic acid 400 μg/day; (6) stop smoking and exposure to any toxic substances; (7) establish a healthy weight and lifestyle; (8) get help with exposure to any violence; and (9) get mentally healthy (Stephenson et al., 2018).

“A sharper focus on intervention before conception is needed to improve maternal and child health and reduce the growing burden of non-communicable disease. Alongside continued efforts to reduce smoking, alcohol and obesity in the population, we call for heightened awareness of preconception health, particularly regarding diet and nutrition. Importantly health professionals should be alerted to ways of identifying women who are planning a pregnancy” (Stephenson et al., 2018).

Published research has demonstrated the important role illness uncertainty plays as an independent contributor to anxiety in women who have Overt POI (Davis et al., 2010). Diabetes self-management education helps people with diabetes incorporate knowledge and navigate treatment decisions as well as establish new and healthier activities known to improve health outcomes (Powers et al., 2016; Chester et al., 2018). This case report demonstrates there is a similar need to develop certified POI Health Coach Educators (Jordan et al., 2015). Women who have Overt POI would benefit from a source of comprehensive guidance which is based on evidence.

Also, there is a need to maintain bone health while attempting conception. The best evidence supports the use of the NIH P-HRT regimen in this regard (Popat et al., 2014). As noted above, taking the NIH P-HRT regimen is known to permit pregnancy to occur (Nelson, 2009). Published expert opinion recommends women who desire to conceive take the following approach: (1) Keep a menstrual calendar while taking the NIH P-HRT regimen. (2) To assure sperm are always present in the event of an unpredictable ovulation have intercourse two or three times a week. (3) Take a pregnancy test if the menstrual period is late. (4) Notify the clinical team about the pregnancy and stop taking the P-HRT. (5) Allow time for conception to occur (up to 3 years in view of the reduced fecundity). (6) While attempting to conceive, make plans for the future in the event no birth takes place (Nelson, 2009).

Finally, as noted, there is no published evidence to support the use of any strategy to monitor follicle development or intervene with any treatment to increase pregnancy rates in women who have Overt POI (Fraison et al., 2019). However, there are specific situations in which monitoring and intervention may be indicated. For example, timing of ovulation becomes critical in the case of severe male factor parameters necessitating donor sperm. Similarly, for some couples having sexual intercourse 2 or 3 times a week is quite difficult. Infertility is known to be associated with a high incidence of sexual dysfunction in general (Riazi et al., 2020). Specifically, as compared with controls, women who have Overt POI, despite taking physiologic estradiol replacement score significantly lower on a validated sexual function scale (Kalantaridou et al., 2008). Presently, there are many options for women to employ in an attempt to time ovulation (Owen, 2013; Su et al., 2017). Cervical mucus monitoring deserves evaluation as a simple, free, readily available method which is supported by published evidence to improve pregnancy rates in the general population (Evans-Hoeker et al., 2013). None of these methods have been proven to be effective in women who have Overt POI. There is a need for prospective study to generate validated protocols for predicting ovulation in these women. Such a protocol might improve pregnancy rates by permitting timing of intercourse, donor insemination, intrauterine insemination, natural cycle *in vitro* fertilization (IVF), or intracytoplasmic sperm injection (ICSI) (Farquhar and Marjoribanks, 2018).

## Social Media

POI is a rare disorder. For those affected, finding reliable information on rare diseases is a major challenge. Limited data is available regarding the use of information sources for various rare diseases, or how the usefulness of the information is evaluated by patients and their families (Litzkendorf et al., 2020). One qualitative study reported patients with various rare diseases and their close relatives considered the internet as the most important and widespread information source. They considered searching online an expedient and practical manner for gathering information. Online information sometimes was insufficiently detailed to satisfy their needs.

Self-help associations and specialized clinicians help in filling the information gaps (Litzkendorf et al., 2020). Facebook is used commonly as a source of health-related information. However, social media involves the risk of getting misinformation and the risk of sharing of confidential health information. One online cross-sectional study surveyed patients with psoriasis to determine the relevance and suitability of Facebook as a source of disease-related information (Schuster et al., 2020). The study found Facebook helped them cope with psoriasis to a degree, but a majority (60%) reported finding unreliable information regarding the disorder. The authors concluded Facebook is a relevant source of health information for patients who have psoriasis. However, the quality of information was deemed inadequate and is an area in need of improvement (Schuster et al., 2020).

There is a need to improve the quality of information about POI on Facebook. One specific closed group with over 1,500 members has a Facebook page working toward this goal. The group, *My Family Cares About Primary Ovarian Insufficiency and Early Menopause*, has been developed and maintained by the Conover Foundation (My Family Cares about Primary Ovarian Insufficiency and Early Menopause, n.d.). The page provides a health coach serving as a POI Clinical Navigator as well as an experienced physician/scientist to curate the page. The physician/scientist has 30 years of experience working with women who have dealt with the difficult life transitions which come with a diagnosis of POI/EM. The goal of the group is to maintain coherent discussion threads, provide peer-reviewed resources, and assure information shared is based on the best available medical evidence. On this Facebook page, women can read stories of international women expressing views regarding their experiences with POI/EM. They are able to share stories about getting the diagnosis, symptoms they have experienced, mention long-term health effects, describe treatments, report experiences with health services and health practitioners, and express their assessment regarding the impact POI/EM has had in their life and in their personal relationships. These are just a few aspects of the information women can find there. The page provides links to a comprehensive range of resources. When needed, the page offers a more personalized approach using videoconference to connect, educate, and coach clients with the help of a trained POI clinical navigator.

## Genetic Causes

Recent evidence has increased dramatically the number of candidate loci for POI (Huhtaniemi et al., 2018; Bestetti et al., 2019). Several new candidate genes account for mechanisms of DNA replication and repair. The systematic use of the Next-Generation Sequencing (NGS) approach with targeted exome sequencing has revealed frequent oligogenic involvement, mainly in cases with earlier onset of POI (Rossetti et al., 2017, in press; Cattoni et al., 2020).

### Turner Syndrome X Chromosome Defects

Both X-linked and autosomal genes are involved in POI. Some are part of a syndrome and some lead to isolated POI (Rossetti et al., 2017). Both familial and sporadic forms of POI are associated with X chromosome abnormalities. Turner syndrome, Turner mosaic, triple X syndrome, partial X deletions, isochromosomes, and translocations between X chromosome and autosomes are well-known examples (Goswami et al., 2003; Toniolo, 2006). Either complete or partial loss of one X chromosome may result in Turner syndrome. The Turner syndrome phenotype includes POI, short stature, and various somatic abnormalities. Turner syndrome has an incidence of about 1:2,500 live female births (Rossetti et al., 2017).

### Fragile X-Associated Primary Ovarian Insufficiency (*FMR1*)

Premutation in *FMR1* is an established genetic cause of Overt POI, termed fragile X-associated primary ovarian insufficiency (FXPOI) (Sherman, 2000). Impaired fertility is an important complication of FXPOI (Allen et al., 2007; Streuli et al., 2009). A premutation in FMRI is found in about 11% of women who have a family member with POI and in about 3.2% of women who have isolated POI (Sherman et al., 2007). A premutation is defined as 55–199 expanded CGG repeats located in the 5′ untranslated region (UTR) of the X-linked gene, *FMR1*. About 1/300 women carry a premutation (Hunter et al., 2014). Of women who have a premutation, overt FXPOI occurs in about 20% (Sherman, 2000; Sullivan et al., 2005; De Caro et al., 2008).

There are three risk factors associated with the development of FXPOI. Firstly, CGG repeat size has a non-linear relationship with the risk for FXPOI (Spath et al., 2011). The risk for FXPOI is about 10% for women with 55–79 repeats, 32% for women with 80–100 repeats, and 16% for women with more than 100 repeats (Allen et al., 2007). Earlier onset of the disorder is associated with women who have 80–100 repeats, in some cases even during adolescence (De Caro et al., 2008). Secondly, the risk of the disorder increases if there is a family history of early menopause (Spath et al., 2011). This indicates there are background genetic variants which contribute to the risk. Smokers have an earlier menopause, and there is a similarity in women who have a premutation (Allen et al., 2007).

For woman carrying a premutation, genetic counseling is essential. They are at risk of having a child with fragile X syndrome in addition to the risk of FXPOI and its clinical consequences. There is also risk for fragile X-associated tremor/ataxia syndrome (FXTAS), which is a neurodegenerative disorder developing at approximately age 60 (Hagerman et al., 2001; Hawkins and Matzuk, 2008; Harris et al., 2016). Women have a lifetime prevalence of FXTAS of 6–18% (Wheeler et al. 2014).

Basic science as well as clinical evidence suggest a novel mechanism for FXPOI. Little is known regarding the ovarian histopathology in women with FXPOI. One study with a small number of cases showed ovarian histology and follicle number similar to controls (Chang et al., 2011). Therefore, the mechanism of FXPOI appears to be one of follicle dysfunction rather than depletion. Similar findings have been reported in mouse models of FXPOI. Mice carrying an *FMR1* premutation were reported to have a normal complement of primordial follicles (Conca Dioguardi et al., 2016). Thus, FXPOI seems likely to be a disorder of impaired follicle function.

In one case report, a young woman who had FXPOI conceived without medical intervention while on the NIH P-HRT regimen (Fink et al., 2018). She terminated the pregnancy as prenatal genetic testing showed a full mutation in *FMR1* in the fetus. After that, she conceived two more pregnancies without medical intervention while on the same P-HRT regimen, one in 2013 and one in 2016. Both pregnancies and deliveries were unremarkable. Despite her history of having had two prior pregnancies after the diagnosis of FXPOI, a physician at an IVF clinic suggested she proceed to egg donation and quoted a current chance of conception of <0.1%. This remarkable report about fertility in FXPOI while on the NIH P-HRT regimen suggests a need for more research into this approach to optimize fertility in women who have FXPOI, especially in view of the above evidence supporting follicle dysfunction rather than low number of follicles as the mechanism.

### Autoimmune Polyendocrinopathy Syndrome Type I

Autoimmune polyendocrinopathy syndrome type I (*APS1*), made manifest by mutations in the autoimmune regulator gene (*AIRE*, MIM ^*^607358), is a syndrome confirmed by the presence of two of these important clinical disorders: Addison's disease (AD), hypoparathyroidism, and chronic mucocutaneous candidiasis. Generally, AD usually presents as a child or young adult. The syndrome may be associated with primary hypogonadism, pernicious anemia, alopecia, malabsorption, or chronic active hepatitis (Rossetti et al., 2017).

### Gonadotropin Receptors FSH and LH (*FSHR, LHCGR*)

FSH and LH receptors belong to the G-protein-coupled receptors (GPCRs) family. These receptors regulate reproductive hormone signaling by interacting with FSH and LH in both men and women. Loss-of-function mutations in these receptors result in resistance to gonadotropin stimulation and present with hypogonadotropic hypogonadism (Themmen and Huhtaniemi, 2000).

### Blepharophimosis, Ptosis, Epicanthus Inversus Syndrome

Blepharophimosis, ptosis, epicanthus inversus syndrome (BPES) is an eyelid malformation characterized by BPES and telecanthus. The condition is autosomal dominant. BPES is considered type I when present in association with POI. Type II BPES is not associated with POI. Forkhead transcription factor L2 (*FOXL2*, MIM ^*^605597) mutations are associated with BPES (Crisponi et al., 2001).

### Galactosemia (*GALT*)

The normal ovary requires normal galactose metabolism. In galactosemia, galactose metabolism is abnormal due to a deficiency of the enzyme galactose-1-phosphatase uridyltransferase (GALT). The estimated incidence in Europe and North America is 1:30,000–1:50,000 (Rubio-Gozalbo et al., 2010). Organs with high GALT activity such as the ovary, liver, kidney, and heart are prone to the worst malfunction. Most women (80–90%) with *GALT* mutations which are homozygous, show a severe phenotype and exhibit POI (Rubio-Gozalbo et al., 2010).

### Pseudo-Hypoparathyroidism Type 1a

Although pseudo-hypoparathyroidism type 1a (PHP1a, MIM #103580) is a renal resistance to parathyroid hormone (PTH), causing hypocalcemia and hyperphosphatemia, the disorder is in fact characterized by resistance to other hormones to include gonadotropins. Thus, delayed or incomplete gonadal function with associated amenorrhea, oligomenorrhea, or infertility is commonly part of the clinical disorder (Mantovani and Spada, 2006). Resistance to other hormones such as thyroid-stimulating hormone (TSH) or growth-hormone-releasing hormone (GHRH) may be present. The clinical disorder known as Albright hereditary osteodystrophy is part of the syndrome.

### Progressive External Ophthalmoplegia (*POLG*)

The POLG gene encodes the enzyme that synthesizes mitochondrial DNA and corrects errors. Dysfunction in this gene results in a severe progressive multisystem disorder. The disorder includes parkinsonian symptoms as well as POI. These are not typical of mitochondrial disease (Luoma et al., 2004; Pagnamenta et al., 2006).

### Ovarioleukodystrophies

By definition, ovarioleukodystrophies are genetic disorders in which POI is associated with neurological disorders characterized by abnormalities in the white matter of the central nervous system (Mathis et al., 2008).

### Ataxia Telangiectasia

Mutation in the ataxia telangiectasia gene (ATM) is associated with primordial germ cell development defects. This gene encodes a cell-cycle checkpoint kinase. The gene contributes to the cellular response to DNA damage and processes DNA strand breaks. These occur with meiosis, immune system maturation, and telomere maintenance (Kastan and Bartek, 2004).

### Demirhan Syndrome (*BMPR1B*)

Demirhan syndrome is a subtype of chondrodysplasia associated with amenorrhea, hypogonadism, and genital anomalies. A mutation in BMPR1B causes the disorder. This gene encodes bone morphogenetic protein receptor 1B (Demirhan et al., 2005).

### Bloom Syndrome (*BLM*)

Bloom syndrome is an autosomal recessive disorder that is rare. Mutations in the gene coding for the DNA helicase *BLM* cause the disorder. The gene encodes the DNA helicase *BLM* (MIM #604610). Mutations lead to genomic instability. The disorder involves hypogonadism in both sexes, short stature, moderate immunodeficiency, increased cancer rate, and distinctive skin rashes on sun-exposed areas (Arora et al., 2014).

### Werner Syndrome (*WRN*)

Recessive mutations in the *WRN* gene cause Werner syndrome, a form of adult progeria. WRN encodes a DNA helicase. This is a form of adult progeria. The disorder is characterized by atrophic gonads, scleroderma-like skin, cataracts, increased cancer rate, and premature arteriosclerosis (Rossi et al., 2010).

### GAPO Syndrome (*ANTXR1*)

GAPO is a syndrome of premature aging associated with POI. It is characterized by distinctive facial features, optic atrophy, growth retardation, alopecia, and optic atrophy. Ovarian histology reveals extensive deposition of hyaline extracellular material and depletion of ovarian follicles (Benetti-Pinto et al., 2016). *GAPO* is caused by recessive mutations in a gene involved in cell adhesion and migration, *ANTXR1*.

### Bone Morphogenetic Protein 15

Bone morphogenetic protein 15 (BMP15) (MIM ^*^300247) is a growth/differentiation factor specific to oocytes. The factor is involved in many cellular processes, including follicular development (Persani et al., 2011). The condition may present as either primary or secondary amenorrhea. Mutations in this gene account for the mechanism of POI in about 1.5–15% of cases (Persani et al., 2014).

## Moving Forward

POI is best managed by integrated personal care to address the spectrum of these women's clinical needs. Social media combined with a digital and centralized approach to health managed by an experienced team promises to improve this situation. Insights into molecular pathogenetic mechanisms are needed to permit prevention and early diagnosis. Most cases of spontaneous primary ovarian insufficiency are idiopathic. As a rare disease, POI diagnosis presents difficult problems in management. A strategy to improve care and facilitate research is needed. A comprehensive approach for managing POI is long overdue. A Clinical POI Navigator could be a valuable addition to improving the quality of care for these women and optimizing their overall health and fertility. A properly managed cloud-based system could connect women with POI, a POI navigator, primary care providers, and investigators who have the requisite knowledge and expertise (Martin et al., 2017).

In sum, there is a need for a new approach to POI (Cooper et al., 2011). There are major gaps in knowledge regarding this disorder's medical and psychosocial management, psychosocial effects, and natural history. An international disease registry and research consortium formed under an umbrella organization's guidance would provide a pathway to increase basic and clinical knowledge about the condition. Such a consortium and patient registry would also provide clinical samples and clinical data to define the disorder's specific mechanisms further. The effort would combine a patient registry and a community-based participatory research structure. The systematic application of a comprehensive NGS panel for genetic diagnoses may permit early diagnosis in young women who come from a family at higher risk of POI. This would facilitate fertility preservation for women identified as at high risk for POI.

## Summary and Conclusions

Overt POI presents with oligo/amenorrhea and estradiol deficiency symptoms. Laboratory investigation reveals hypergonadotropic hypogonadism. The condition is a chronic disorder with increased morbidity and long-term mortality related to the reduced bone mineral density and increased risk of cardiovascular disease related to estradiol deficiency. The associated infertility is life altering. Here, we report the case of a young woman with Overt POI who had a successful pregnancy. She conceived on the eighth cycle of monitoring follicle development while taking the NIH P-HRT to reduce serum LH levels. There is a need for increased awareness regarding the fertility potential of women who have POI. The pathophysiology of follicle dysfunction has been defined as inappropriate luteinization of Graafian follicles. P-HRT has been demonstrated to lower LH levels in these women and would be expected to improve ovulation rates, as demonstrated in this case report.

## Data Availability Statement

The datasets for this article are not publicly available because family consents to share data publicly was not allowed. Requests to access the datasets should be directed to Dr. Muhammad Imran Naseer, mimrannaseer@yahoo.com.

## Ethics Statement

Written informed consent was obtained from the minor(s)' legal guardian/next of kin for the publication of any potentially identifiable images or data included in this article.

## Author Contributions

KP helped develop the focus for the report and contributed content regarding managing POI. HS contributed the case report. LP contributed intellectual content regarding the pathophysiology of POI and the genetic mechanisms. LN conceived the purpose for the report and integrated the content to create the first draft. All authors approved the manuscript for submission.

## Conflict of Interest

LN is employed by companies William Brown Consulting, LLC and My Family Cares, LLC. The remaining authors declare that the research was conducted in the absence of any commercial or financial relationships that could be construed as a potential conflict of interest.

## References

[B1] Allen E. G. Sullivan A. K. Marcus M. Small C. Dominguez C. Epstein M. P.. (2007). Examination of reproductive aging milestones among women who carry the FMR1 premutation. Hum. Reprod. 22, 2142–2152. 10.1093/humrep/dem148 17588953

[B2] Arora H. Chacon A. H. Choudhary S. McLeod M. P. Meshkov L. Nouri K.. (2014). Bloom syndrome. Int. J. Dermatol. 53, 798–802. 10.1111/ijd.12408 24602044

[B3] Benetti-Pinto C. L. Ferreira V. Andrade L. Yela D. A. De Mello M. P. (2016). GAPO syndrome: a new syndromic cause of premature ovarian insufficiency. Climacteric J. Int. Menopause Soc. 19, 594–598. 10.1080/13697137.2016.1200551 27426988

[B4] Bestetti I. Castronovo C. Sironi A. Caslini C. Sala C. Rossetti R.. (2019). High-resolution array-CGH analysis on 46, XX patients affected by early onset primary ovarian insufficiency discloses new genes involved in ovarian function. Hum. Reprod. 34, 574–583. 10.1093/humrep/dey389 30689869 PMC6389867

[B5] Bracewell-Milnes T. Srdjan Saso H. A. Thum M. (2018). A systematic review investigating psychosocial aspects of egg sharing in the United Kingdom and their potential effects on egg donation numbers. Hum. Fertil. 21, 163–173. 10.1080/14647273.2017.1329554 28549399

[B6] Canonico M. Plu-Bureau G. Lowe G. D. O. Scarabin P. (2008). Hormone replacement therapy and risk of venous thromboembolism in postmenopausal women: systematic review and meta-analysis. BMJ 336, 1227–1231. 10.1136/bmj.39555.441944.BE 18495631 PMC2405857

[B7] Cattoni A. Spano A. Tulone A. Boneschi A. Masera N. Maitz S.. (2020). The Potential Synergic Effect of a Complex Pattern of Multiple Inherited Genetic Variants as a pathogenic factor for ovarian dysgenesis: a case report. Front. Endocrinol. 11:540683. 10.3389/fendo.2020.540683 33101191 PMC7545356

[B8] Chang M. C. DeCaro J. J. Zheng M. Gearing M. Shubeck L. Sherman S. L.. (2011). Ovarian histopathological and ubiquitin-immunophenotypic features in fragile X-associated primary ovarian insufficiency: a study of five cases and selected controls. Histopathology 59, 1018–1023. 10.1111/j.1365-2559.2011.03959.x 22007616 PMC3220768

[B9] Chester B. Stanely W. G. Geetha T. (2018). Quick guide to type 2 diabetes self-management education: creating an interdisciplinary diabetes management team. Diabet. Metabol. Syndrome Obes. 11, 641–645. 10.2147/DMSO.S178556 30410376 PMC6199222

[B10] Committee Opinion No. 698: hormone therapy in primary ovarian insufficiency (2017). Obstetr. Gynecol. 129, e134–41. 10.1097/AOG.0000000000002044 28426619

[B11] Conca Dioguardi C. Uslu B. Haynes M. Kurus M. Gul M. Miao D.. (2016). Granulosa cell and oocyte mitochondrial abnormalities in a mouse model of fragile x primary ovarian insufficiency. Mol. Hum. Reprod. 22, 384–396. 10.1093/molehr/gaw023 26965313 PMC4884918

[B12] Cooper A. R. Baker V. L. Sterling E. W. Ryan M. E. Woodruff T. K. Nelson L. M. (2011). The time is now for a new approach to primary ovarian insufficiency. Fertil. Steril. 95, 1890–1897. 10.1016/j.fertnstert.2010.01.016 20188353 PMC2991394

[B13] Covington S. N. Hillard P. J. Sterling E. W. Nelson L. M. (2011). A family systems approach to primary ovarian insufficiency. J. Pediatr. Adolesc. Gynecol. 24, 137–141. 10.1016/j.jpag.2010.12.004 21269850 PMC3094722

[B14] Crisponi L. Deiana M. Loi A. Chiappe F. Uda M. Amati P.. (2001). The putative forkhead transcription factor FOXL2 is mutated in blepharophimosis/ptosis/epicanthus inversus syndrome. Nat. Genet. 27, 159–166. 10.1038/84781 11175783

[B15] Davis M. Ventura J. L. Wieners M. Covington S. N. Vanderhoof V. H. Ryan M. E.. (2010). The psychosocial transition associated with spontaneous 46,XX primary ovarian insufficiency: illness uncertainty, stigma, goal flexibility, and purpose in life as factors in emotional health. Fertil. Steril. 93, 2321–2329. 10.1016/j.fertnstert.2008.12.122 19243752 PMC3013503

[B16] De Caro J. J. Dominguez C. Sherman S. L. (2008). Reproductive health of adolescent girls who carry the FMR1 premutation: expected phenotype based on current knowledge of fragile x-associated primary ovarian insufficiency. Ann. N. Y. Acad. Sci. 1135, 99–111. 10.1196/annals.1429.029 18574214

[B17] Demirhan O. Turkmen S. Schwabe G. C. Soyupak S. Akgul E. Tastemir D.. (2005). A homozygous BMPR1B mutation causes a new subtype of acromesomelic chondrodysplasia with genital anomalies. J. Med. Genet. 42, 314–317. 10.1136/jmg.2004.023564 15805157 PMC1736042

[B18] Driscoll M. A. Davis M. C. Aiken L. S. Yeung E. W. Sterling E. W. Vanderhoof V.. (2016). Psychosocial vulnerability, resilience resources, and coping with infertility: a longitudinal model of adjustment to primary ovarian insufficiency. Ann. Behav. Med. 50, 272–284. 10.1007/s12160-015-9750-z 26637185

[B19] Evans-Hoeker E. Pritchard D. A. Long D. L. Herring A. H. Stanford J. B. Steiner A. Z. (2013). Cervical mucus monitoring prevalence and associated fecundability in women trying to conceive. Fertil. Steril. 100, 1033–1038. 10.1016/j.fertnstert.2013.06.002 23850303 PMC3787999

[B20] Farquhar C. Marjoribanks J. (2018). Assisted reproductive technology: an overview of cochrane reviews. Cochr. Datab. Syst. Rev. 8:CD010537. 10.1002/14651858.CD010537.pub5 30117155 PMC6953328

[B21] Fink D. A. Nelson L. M. Pyeritz R. Johnson J. Sherman S. L. Cohen Y.. (2018). Fragile X associated primary ovarian insufficiency (FXPOI): case report and literature review. Front. Genet. 9:529. 10.3389/fgene.2018.00529 30542367 PMC6278244

[B22] Fraison E. Crawford G. Casper G. Harris V. Ledger W. (2019). Pregnancy following diagnosis of premature ovarian insufficiency: a systematic review. Reprod. Biomed. Online 39, 467–476. 10.1016/j.rbmo.2019.04.019 31279714

[B23] Gordon C. M. Kanaoka T. Nelson L. M. (2015). Update on primary ovarian insufficiency in adolescents. Curr. Opin. Pediatr. 27, 511–519. 10.1097/MOP.0000000000000236 26087426

[B24] Goswami R. Goswami D. Kabra M. Gupta N. Dubey S. Dadhwal V. (2003). Prevalence of the triple X syndrome in phenotypically normal women with premature ovarian failure and its association with autoimmune thyroid disorders. Fertil. Steril. 80, 1052–1054. 10.1016/S0015-0282(03)01121-X 14556833

[B25] Groff A. A. Covington S. N. Halverson L. R. Fitzgerald O. R. Vanderhoof V. Calis K.. (2005). Assessing the emotional needs of women with spontaneous premature ovarian failure. Fertil. Steril. 83, 1734–1741. 10.1016/j.fertnstert.2004.11.067 15950644

[B26] Hagerman R. J. Leehey M. Heinrichs W. Tassone F. Wilson R. Hills J.. (2001). Intention tremor, parkinsonism, and generalized brain atrophy in male carriers of Fragile X. Neurology 57, 127–130. 10.1212/wnl.57.1.127 11445641

[B27] Harris J. G. Bingham C. A. Morgan E. M. (2016). Improving care delivery and outcomes in pediatric rheumatic diseases. Curr. Opin. Rheumatol. 28, 110–116. 10.1097/BOR.0000000000000257 26780426 PMC5175576

[B28] Hawkins S. M. Matzuk M. M. (2008). The menstrual cycle: basic biology. Ann. N. Y. Acad. Sci. 1135, 10–18. 10.1196/annals.1429.018 18574203 PMC2913133

[B29] Hipp H. S. Charen K. H. Spencer J. B. Allen E. G. Sherman S. L. (2016). Reproductive and gynecologic care of women with fragile X primary ovarian insufficiency (FXPOI). Menopause 23, 993–999. 10.1097/GME.0000000000000658 27552334 PMC4998843

[B30] Hubayter Z. R. Popat V. Vanderhoof V. H. Ndubizu O. Johnson D. Mao E.. (2010). A prospective evaluation of antral follicle function in women with 46,XX spontaneous primary ovarian insufficiency. Fertil. Steril. 94, 1769–1774. 10.1016/j.fertnstert.2009.10.023 19939372 PMC2888894

[B31] Huhtaniemi I. Hovatta O. La Marca A. Livera G. Monniaux D. Persani L.. (2018). Advances in the molecular pathophysiology, genetics, and treatment of primary ovarian insufficiency. Trends Endocrinol. Metab. 29, 400–419. 10.1016/j.tem.2018.03.010 29706485

[B32] Hunter J. Rivero-Arias O. Angelov A. Kim E. Fotheringham I. Leal J. (2014). Epidemiology of Fragile X syndrome: a systematic review and meta-analysis. Am. J. Med. Genet. A 164A, 1648–1658. 10.1002/ajmg.a.36511 24700618

[B33] Jordan M. Wolever R. Q. Lawson K. Moore M. (2015). National training and education standards for health and wellness coaching: the path to national certification. Glob. Adv. Health Med. 4, 46–56. 10.7453/gahmj.2015.039 25984418 PMC4424935

[B34] Kalantaridou S. N. Vanderhoof V. H. Calis K. A. Corrigan E. C. Troendle J. F. Nelson L. M. (2008). Sexual function in young women with spontaneous 46,XX primary ovarian insufficiency. Fertil. Steril. 90, 1805–1811. 10.1016/j.fertnstert.2007.08.040 17961560 PMC2592535

[B35] Kastan M. B. Bartek J. (2004). Cell-cycle checkpoints and cancer. Nature 432, 316–323. 10.1038/nature03097 15549093

[B36] Litzkendorf S. Frank M. Babac A. Rosenfeldt D. Schauer F. Hartz T.. (2020). Use and importance of different information sources among patients with rare diseases and their relatives over time: a qualitative study. BMC Public Health 20:860. 10.1186/s12889-020-08926-9 32503483 PMC7275578

[B37] Livneh H. (2019). The use of generic avoidant coping scales for psychosocial adaptation to chronic illness and disability: a systematic review. Health Psychol. Open 6:2055102919891396. 10.1177/2055102919891396 31839978 PMC6896135

[B38] Luoma P. Melberg A. Rinne J. O. Kaukonen J. A. Nupponen N. N. Chalmers R. M.. (2004). Parkinsonism, premature menopause, and mitochondrial DNA polymerase gamma mutations: clinical and molecular genetic study. Lancet 364, 875–882. 10.1016/S0140-6736(04)16983-3 15351195

[B39] Mantovani G. Spada A. (2006). Mutations in the Gs alpha gene causing hormone resistance. Best practice and research. Clin. Endocrinol. Metab. 20, 501–513. 10.1016/j.beem.2006.09.001 17161328

[B40] Martin L. A. Porter A. G. Pelligrini V. A. Schnatz P. F. Jiang X. Kleinstreuer N.. (2017). A design thinking approach to primary ovarian insufficiency. Panminerva Med. 59, 15–32. 10.23736/S0031-0808.16.03259-6 27827529

[B41] Mathis S. Scheper G. C. Baumann N. Petit E. Gil R. van der Knaap M. S.. (2008). The ovarioleukodystrophy. Clin. Neurol. Neurosurg. 110, 1035–1037. 10.1016/j.clineuro.2008.06.002 18678442

[B42] Mishell D. R. Nakamura R. M. Crosignani P. G. Stone S. Kharma K. Nagata Y.. (1971). Serum gonadotropin and steroid patterns during the normal menstrual cycle. Am. J. Obstet. Gynecol. 111, 60–65. 10.1016/0002-9378(71)90927-6 5096358

[B43] Mohammed K. Abu Dabrh A. M. Benkhadra K. Al Nofal A. Carranza Leon B. G. Prokop L. J.. (2015). Oral vs Transdermal Estrogen Therapy and Vascular Events: A Systematic Review and meta-analysis. J. Clin. Endocrinol. Metab. 100, 4012–4020. 10.1210/jc.2015-2237 26544651

[B44] My Family Cares about Primary Ovarian Insufficiency Early Menopause (n.d.). Home [Facebook Page]. Facebook. Available online at: https://www.facebook.com/groups/64513377304 (accessed March 3 2021).

[B45] National Board for Health Wellness Coaching (n.d.). National Board for Health Wellness Coaching. Available online at: https://nbhwc.org/ (accessed January 4 2012).

[B46] Nelson L. M. (2009). Clinical practice. primary ovarian insufficiency. New Eng. J. Med. 360, 606–614. 10.1056/NEJMcp0808697 19196677 PMC2762081

[B47] Nelson L. M. Anasti J. N. Kimzey L. M. Defensor R. A. Lipetz K. J. White B. J.. (1994). Development of luteinized graafian follicles in patients with karyotypically normal spontaneous premature ovarian failure. J. Clin. Endocrinol. Metab. 79, 1470–1475. 10.1210/jcem.79.5.7962345 7962345

[B48] Orshan S. A. Ventura J. L. Covington S. N. Vanderhoof V. H. Troendle J. F. Nelson L. M. (2009). Women with spontaneous 46,XX primary ovarian insufficiency (Hypergonadotropic Hypogonadism) have lower perceived social support than control women. Fertil. Steril. 92, 688–693. 10.1016/j.fertnstert.2008.07.1718 18829005 PMC2734403

[B49] Owen M. (2013). Physiological signs of ovulation and fertility readily observable by women. Linacre Q. 80, 17–23. 10.1179/0024363912Z.0000000005 24845657 PMC6081768

[B50] Pagnamenta A. T. Taanman J. Wilson C. J. Anderson N. E. Marotta R. Duncan A. J.. (2006). Dominant inheritance of premature ovarian failure associated with mutant mitochondrial DNA polymerase gamma. Hum. Reprod. 21, 2467–2473. 10.1093/humrep/del076 16595552

[B51] Persani L. Rossetti R. Cacciatore C. Fabre S. (2011). Genetic defects of ovarian TGF-β-like factors and premature ovarian failure. J. Endocrinol. Invest. 34, 244–251. 10.1007/BF03347073 21297384

[B52] Persani L. Rossetti R. Di Pasquale E. Cacciatore C. Fabre S. (2014). The fundamental role of bone morphogenetic protein 15 in ovarian function and its involvement in female fertility disorders. Hum. Reprod. Update 20, 869–883. 10.1093/humupd/dmu036 24980253

[B53] Popat V. B. Calis K. A. Kalantaridou S. N. Vanderhoof V. H. Koziol D. Troendle J. F.. (2014). Bone mineral density in young women with primary ovarian insufficiency: results of a three-year randomized controlled trial of physiological transdermal estradiol and testosterone replacement. J. Clin. Endocrinol. Metab. 99, 3418–3426. 10.1210/jc.2013-4145 24905063 PMC4154086

[B54] Powers M. A. Bardsley J. Cypress M. Duker P. Funnell M. M.. (2016). Diabetes self-management education and support in type 2 diabetes: a joint position statement of the American Diabetes Association, the American Association of Diabetes Educators, and the Academy of Nutrition and Dietetics. Clin. Diabetes 34, 70–80. 10.2337/diaclin.34.2.70 27092016 PMC4833481

[B55] Renoux C. Dell'aniello S. Garbe E. Suissa S. (2010). Transdermal and oral hormone replacement therapy and the risk of stroke: a nested case-control study. BMJ 340:c2519. 10.1136/bmj.c2519 20525678

[B56] Riazi H. Lotfollahi H. Omani-Samani R. Maroufizadeh S. Montazeri A. (2020). Evaluation of sexual function among infertile women and their sexual self-concept. J. Reprod. Infertil. 21, 291–297. 10.18502/jri.v21i4.4334 33209746 PMC7648864

[B57] Rossetti R. Ferrari I. Bonomi M. Persani L. (2017). Genetics of primary ovarian insufficiency. Clin. Genet. 91, 183–198. 10.1111/cge.12921 27861765

[B58] Rossetti R. Moleri S. Guizzardi F. Gentilini D. Libera L. Marozzi A. (in press). Targeted next-generation sequencing indicates a frequent oligogenic involvement in primary ovarian insufficiency onset. Front. Genet. Accepted. 10.3389/fendo.2021.664645 PMC8600266 34803902

[B59] Rossi M. L. Ghosh A. K. Bohr V. A. (2010). Roles of werner syndrome protein in protection of genome integrity. DNA Repair. 9, 331–344. 10.1016/j.dnarep.2009.12.011 20075015 PMC2827637

[B60] Rossouw J. E. Anderson G. L. Prentice R. L. LaCroix A. Z. Kooperberg C. Stefanick M. L.. (2002). Risks and benefits of estrogen plus progestin in healthy postmenopausal women: principal results from the women's health initiative randomized controlled trial. JAMA 288, 321–333. 10.1001/jama.288.3.321 12117397

[B61] Rubio-Gozalbo M. E. Gubbels C. S. Bakker J. A. Menheere P. P. C. A. Wodzig W. K. W. H. Land J. A. (2010). Gonadal function in male and female patients with classic galactosemia. Hum. Reprod. Update 16, 177–188. 10.1093/humupd/dmp038 19793842

[B62] Schuster B. Ziehfreund S. Biedermann T. Zink A. (2020). Psoriasis 2.0: Facebook Als Quelle Krankheitsbezogener Informationen Für Patienten Mit Psoriasis. J. Dtsch. Dermatol. Ges. 18, 571–581. 10.1111/ddg.14070_g 32519484

[B63] Sherman S. L. (2000). Premature ovarian failure in the Fragile X syndrome. Am. J. Med. Genet. 97, 189–194. 10.1002/1096-8628(200023)97:3<189::AIDAJMG1036>3.0.CO;2-J 11449487

[B64] Sherman S. L. Taylor K. Allen E. G. (2007). “FMR1 premutation: a leading cause of inherited ovarian dysfunction,” in Fragile Sites: New Discoveries and Changing Perspectives, eds Arrieta I. Penagarikano O. Telez M. (Hauppange, NY: Nova Science Publishers, Inc.), 299–320.

[B65] Soave I. Lo Monte G. Marci R. (2013). POI: premature ovarian insufficiency/pregnancy or infertility? North Am. J. Med. Sci. 5:71. 10.4103/1947-2714.106217 23378962 PMC3560145

[B66] Spath M. A. Feuth T. B. Smits A. P. T. Yntema H. G. Braat D. D. M. Thomas C. M. G.. (2011). Predictors and risk model development for menopausal age in fragile X premutation carriers. Genet. Med. 13, 643–650. 10.1097/GIM.0b013e31821705e5 21597380 PMC3132284

[B67] Stephenson J. Heslehurst N. Hall J. Schoenaker D. A. J. M. Hutchinson J. Cade J.. (2018). Before the beginning: nutrition and lifestyle in the preconception period and its importance for future health. Lancet 391, 1830–1841. 10.1016/S0140-6736(18)30311-8 29673873 PMC6075697

[B68] Streuli I. Fraisse T. Ibecheole V. Moix I. Morris M. A. de Ziegler D. (2009). Intermediate and premutation FMR1 alleles in women with occult primary ovarian insufficiency. Fertil. Steril. 92, 464–470. 10.1016/j.fertnstert.2008.07.007 18973899

[B69] Su H. Yi Y. Wei T. Chang T. Cheng C. (2017). Detection of ovulation, a review of currently available methods. Bioeng. Transl. Med. 2, 238–246. 10.1002/btm2.10058 29313033 PMC5689497

[B70] Sullivan A. K. Marcus M. Epstein M. P. Allen E. G. Anido A. E. Paquin J. J.. (2005). Association of FMR1 repeat size with ovarian dysfunction. Hum. Reprod. 20, 402–412. 10.1093/humrep/deh635 15608041

[B71] Sullivan S. D. Sarrel P. M. Nelson L. M. (2016). Hormone replacement therapy in young women with primary ovarian insufficiency and early menopause. Fertil. Steril. 106, 1588–1599. 10.1016/j.fertnstert.2016.09.046 27912889 PMC5137796

[B72] Tao X. Zuo A. Wang J. Tao F. (2016). Effect of primary ovarian insufficiency and early natural menopause on mortality: a meta-analysis. Climacteric 19, 27–36. 10.3109/13697137.2015.1094784 26576012

[B73] Themmen A. P. N. Huhtaniemi I. T. (2000). Mutations of gonadotropins and gonadotropin receptors: elucidating the physiology and pathophysiology of pituitary-gonadal function. Endocr. Rev. 21, 551–583. 10.1210/edrv.21.5.0409 11041448

[B74] Toniolo D. (2006). X-linked premature ovarian failure: a complex disease. Curr. Opin. Genet. Dev. 16, 293–300. 10.1016/j.gde.2006.04.005 16650756

[B75] Wheeler A. C. Bailey D. B. Jr Berry-Kravis E. Greenberg J. Losh M. Mailick M. (2014) Associated Features in Females with an FMR1 Premutation. J. Neurodev. Disord. 6:30. 10.1186/1866-1955-6-30. 25097672 PMC4121434

